# Capture of microparticles by bolus flow of red blood cells in capillaries

**DOI:** 10.1038/s41598-017-05924-7

**Published:** 2017-07-14

**Authors:** Naoki Takeishi, Yohsuke Imai

**Affiliations:** 1 Institute for Frontier Life and Medical Sciences, Kyoto University, Department of Biosystems Science, 53 Shogoin-Kawara-cho, Sakyo, Kyoto 606-8507 Japan; 2 School of Engineering, Tohoku University, 6-6-01 Aoba, Aoba, Sendai 980-8579 Japan

## Abstract

Previous studies have concluded that microparticles (MPs) can more effectively approach the microvessel wall than nanoparticles because of margination. In this study, however, we show that MPs are not marginated in capillaries where the vessel diameter is comparable to that of red blood cells (RBCs). We numerically investigated the behavior of MPs with a diameter of 1 μm in various microvessel sizes, including capillaries. In capillaries, the flow mode of RBCs shifted from multi-file flow to bolus (single-file) flow, and MPs were captured by the bolus flow of the RBCs instead of being marginated. Once MPs were captured, they rarely escaped from the vortex-like flow structures between RBCs. These capture events were enhanced when the hematocrit was decreased, and reduced when the shear rate was increased. Our results suggest that microparticles may be rather inefficient drug carriers when targeting capillaries because of capture events, but nanoparticles, which are more randomly distributed in capillaries, may be more effective carriers.

## Introduction

The flow behavior of microparticles (MPs) is of paramount importance in drug delivery systems targeting capillary districts^1–3^. The behavior of MPs in the microcirculation, therefore, has been widely studied over decades^3, 4^. MPs in blood are subject to hydrodynamic interaction with red blood cells (RBCs), which exhibit axial migration, resulting in MPs appearing primarily in the peripheral layer. This is termed margination, which is the first step in the adhesion of circulating particles to the endothelium. The behavior of platelets has been investigated in *in vivo* experiments using rabbit mesentery, looking at arterioles^5^ and venules^6^ with vessel diameters ranging from 15 to 35 *μ*m. The effects of physical conditions (*e.g*., shear rate) on margination have been systematically investigated using glass tubes^7^ and PDMS channels^8^. These studies provided insight not only into microcirculatory blood flow but also into therapeutic drug carriers. *In vitro* experiments were performed to determine the optimal size/shape of drug carriers to effectively adhere to the vascular wall^9, 10^. For example, Charoenphol *et al*. showed that microspheres (1–10 *μ*m in diameter) more efficiently adhered to the endothelium in microchannels than nanoparticles (≤500 nm in diameter) in blood flow^9^.

Numerical simulations have been also performed to investigate the margination of MPs^11–18^. Müller *et al*.^12^ investigated the effect of the particle size/shape, shear rate, channel width, and volume fraction of RBCs (hematocrit, *Hct*) on margination. Their two-dimensional model showed that large particles (1.83 *μ*m or 0.91 *μ*m in diameter) more efficiently marginated than small particles (0.25 *μ*m in diameter) for various shear rates^12^. Some of the experimental results of MP margination were discussed with numerical results^19, 20^. Lee *et al*.^20^, for example, demonstrated that nanoparticles (200 nm in diameter) randomly distributed in postcapillary venules with a diameter ranging from 15–30 *μ*m, while microparticles (1 *μ*m in diameter) accumulated near the wall.

The experimental and numerical studies mentioned so far have focused on relatively large microvessels. However, MPs are often required to reach capillaries, where the vessel diameter can be comparable to or smaller than RBCs. The flow of RBCs in capillaries has been widely studied by theoretical analyses^21, 22^, *in vivo* experiments^23–27^, and *in vitro* experiments^28–30^. Physical restriction in small capillaries makes RBCs form a single-file line with parachute-shaped deformation, resulting in “*bolus flow*” with vortex-like streamlines between RBCs^21, 22, 31, 32^. Hence, it remains unclear whether the behavior of MPs in such capillaries can be formulated in the same context as margination in relatively large microvessels.

In this study, we numerically investigated the flow of MPs with a diameter of 1 *μ*m for various sizes of microvessels, including capillaries. Our results demonstrated that MPs were not marginated in capillaries, but captured in plasma spaces between RBCs. Once MPs were captured, they rarely escaped from the vortex-like flow between RBCs. We also examined the effect of *Hct* and shear rate on this capture event.

## Results

### Capture event in bolus flow

The flow of MPs was investigated for various vessel diameters ranging from *D* = 8 *μ*m to 22 *μ*m, where the diameter of MPs was *d* ^*P*^ = 1 *μ*m, and the major diameter of RBCs was *d* ^*R*^ = 8 *μ*m. First, we focused on a shear rate of $\dot{\gamma }$ = 167 s^−1^ and an RBC volume fraction of *Hct* = 0.2. The shear rate can be normalized to form the capillary number *Ca* = $\mu \dot{\gamma }{d}^{R}\mathrm{/2}{G}_{s}^{R}$, such that $\dot{\gamma }$ = 167 s^−1^ corresponds to *Ca* = 0.2, where *μ* is the viscosity of plasma, ${G}_{s}^{R}$ is the surface shear elastic modulus of RBCs. For more details, see Methods.

**Figure 1:**
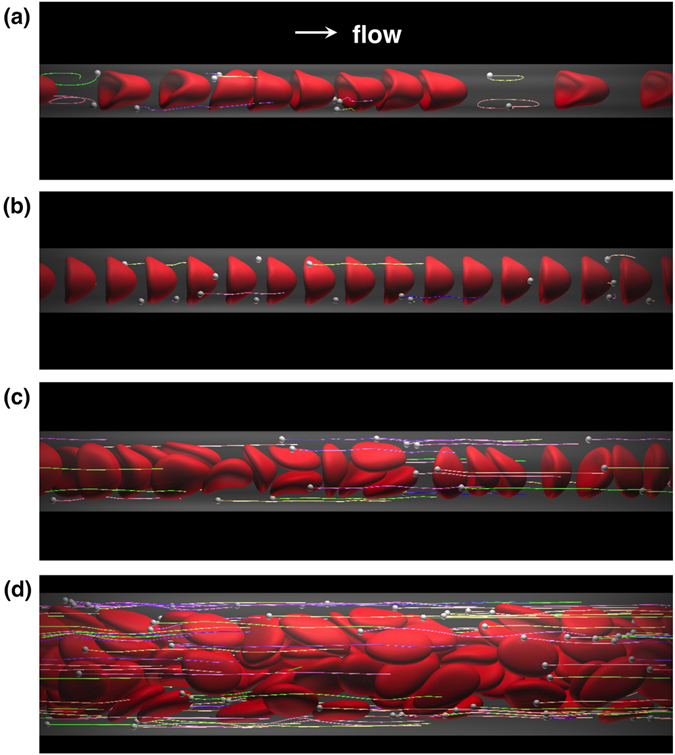
Snapshots of the flow of MPs and RBCs for *Hct* = 0.2 and *Ca* = 0.2 in microvessels with diameters (**a**) *D* = 8 *μ*m, (**b**) 10 *μ*m, (**c**) 12 *μ*m, and (**d**) 22 *μ*m. The flow is from left to right. Lines show particle trajectories. See also Supplemental Movies S1 to S4.

Snapshots of numerical results are shown in Fig.1. In the smallest microvessel (*D* = 8 *μ*m), RBCs formed a single-file line, resulting in bolus flow with vortex-like streamlines^21, 22, 31, 32^. Some MPs were then captured in the plasma spaces between RBCs and circulated in the vortex (Fig. 1a; see also Supplemental Movie S1). Hereafter, we call this phenomenon a “capture event”. Bolus flow in *D* = 8 *μ*m was not so stable, and RBCs sometimes showed clustering. When the vessel diameter increased to *D* = 10 *μ*m, RBCs underwent a more stable single-file motion, in that the size of the spaces between RBCs remained constant, and captured MPs in the bolus flow rarely escaped from the vortex (Fig. 1b; see also Supplemental Movie S2). When the vessel diameter further increased to *D* = 12 *μ*m, the flow mode sifted to a transition state, where single-file and multi-file motions coexist^33, 34^, and most MPs were marginated in cell-depleted peripheral layer (CDPL) (Fig. 1c; see also Supplemental Movie S3). In the largest microvessel (*D* = 22 *μ*m), RBCs formed complete multi-file flow^35, 36^. Most MPs were also marginated, but only after a longer period of time (Fig. 1d; see also Supplemental Movie S4).

**Figure 2:**
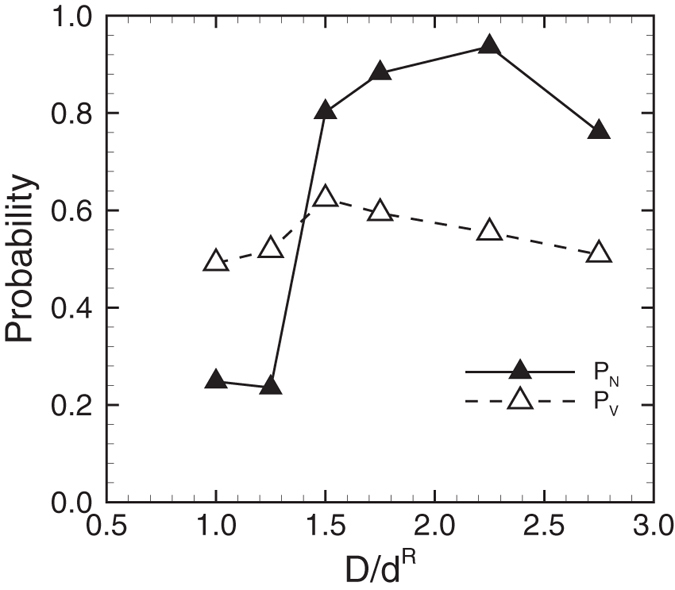
Number probability of MPs in CDPL, *P*_*N*_, and volume fraction of CDPL, *P*_*V*_, for *Hct* = 0.2 and *Ca* = 0.2. The microvessel diameter is normalized by that of RBCs, *D*/*d* ^*R*^.

Figure 2 compares the number probability of MPs in CDPL (*P*_*N*_ = *N*_*M*_/*N*_*T*_) with the volume fraction of CDPL (*P*_*V*_ = *V*_*CDPL*_/*V*_*T*_), where *N*_*M*_ is the number of MPs in CDPL, *N*_*T*_ is the total number of MPs, *V*_*CDPL*_ is the volume of CDPL, and *V*_*T*_ is the total volume of the computational domain. When MPs are randomly distributed in the vessel, the number probability should be the same value as the volume fraction. However, when MPs are marginated, the number probability becomes larger than the volume fraction. The number probability was larger than the volume fraction for *D* ≥ 12 *μ*m, so these cases could be defined as margination. However, because of the capture of MPs in capillary-sized microvessels, the number probability becomes lower than the volume fraction for *D* < 12 *μ*m.

**Figure 3:**
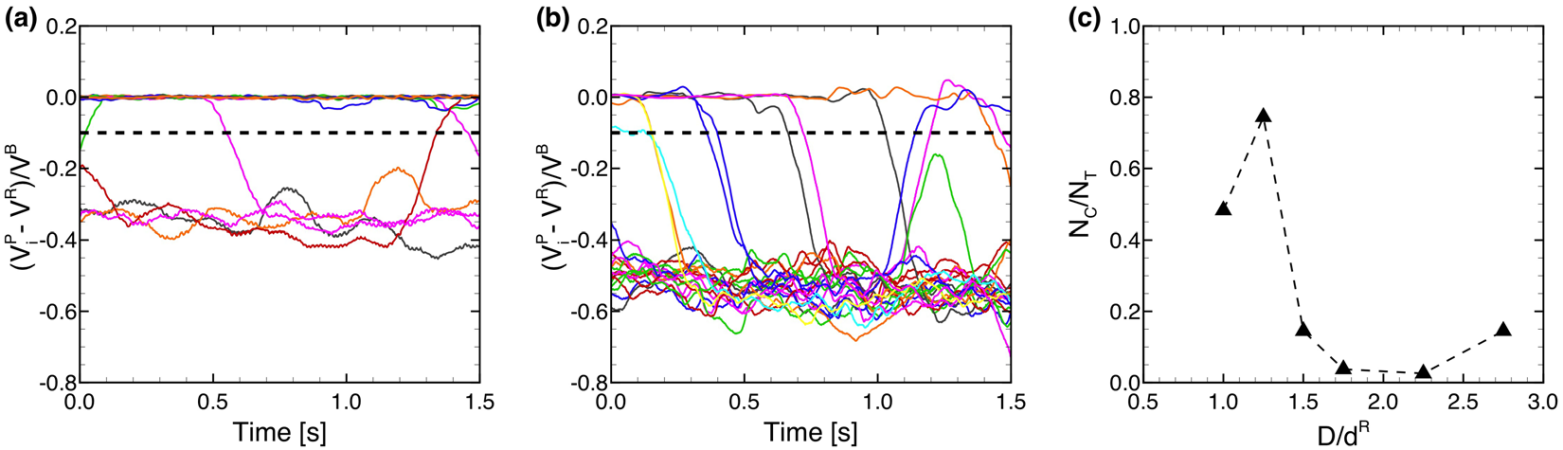
Time history of relative velocity ratio, $({V}_{i}^{P}-{V}^{R})/{V}^{B}$ for (**a**) *D* = 10 *μ*m (18 MPs), and (**b**) 12 *μ*m (24 MPs), where moving-average velocity of each particle (${V}_{i}^{p}$) relative to mean RBC velocity (*V* ^*R*^) is normalized by the whole blood velocity (*V* ^*B*^). The moving-average was conducted for a time period of 0.2 s. Dotted line represents the capture threshold, $({V}_{i}^{P}-{V}^{R})/{V}^{B}$ = −0.1. (**c**) Capture ratio of MPs, where *N*_*C*_ is the number of captured MPs and *N*_*T*_ is the total number of MPs.

To quantify the capture events, a capture ratio was defined. When an MP is captured in the vortex between RBCs, the net velocity of the MP should be the same as the velocity of the RBCs, *i.e*., ${V}_{i}^{P}-{V}^{R}\approx 0$, where the subscript *i* represents the *i*th MP, ${V}_{i}^{P}$ is the moving average of the MP velocity, and *V* ^*R*^ is the mean velocity of the RBCs. The time histories of the relative velocity of each MP in bolus flow (*D* = 10 *μ*m) and transition state (*D* = 12 *μ*m) are shown in Fig. 3a,b, respectively, where the moving average was conducted for a time period of 0.2 s. In bolus flow (Fig. 3a), 13–14 particles were captured with velocities of $({V}_{i}^{P}-{V}^{R})/{V}^{B}\approx 0$, while 4–5 particles were marginated with velocities of $({V}_{i}^{P}-{V}^{R})/{V}^{B}\approx -0.3$, where *V* ^*B*^ is the mean velocity of blood. However, in transition state (Fig. 3b), most MPs were marginated and had velocities of $({V}_{i}^{P}-{V}^{R})/{V}^{B}\approx -0.5$. Thus, we define the capture of MPs as $({V}_{i}^{P}-{V}^{R})/{V}^{B} > -0.1$, and the capture ratio is then defined as *N*_*C*_/*N*_*T*_, where *N*_*C*_ is the number of the captured MPs. The capture ratio is shown in Fig. 3c. The capture ratio was approximately 0.5 for *D*/*d* ^*R*^ = 1.0, increased to 0.8 for *D*/*d* ^*R*^ = 1.25, then suddenly decreased to values less than 0.2 for *D*/*d* ^*R*^ > 1.25.

### Effect of *Hct* on capture ratio

**Figure 4:**
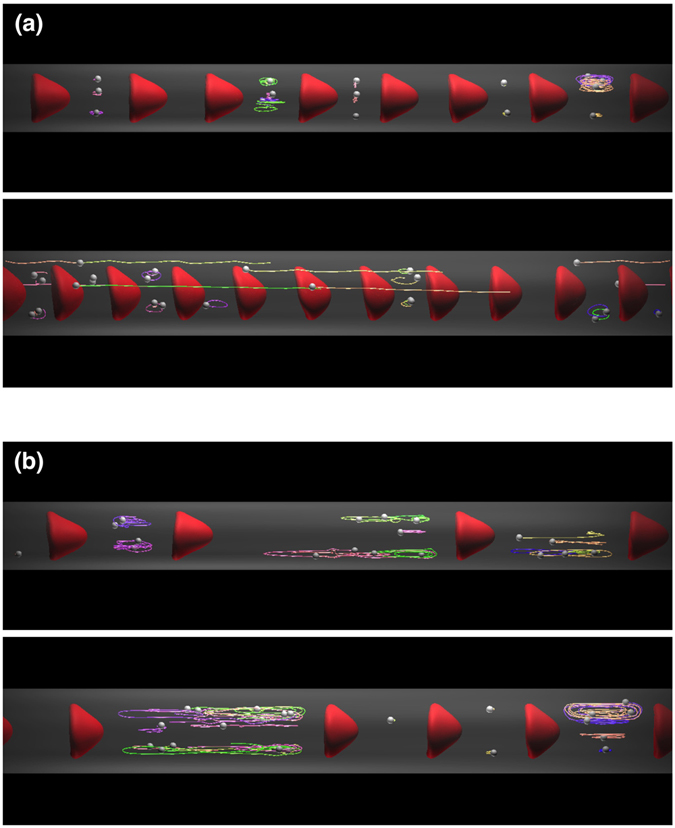
Snapshots of the flow of MPs and RBCs for *Ca* = 0.2 and (**a**) *Hct* = 0.1 and (**b**) 0.05 in capillaries with diameters (top) *D* = 10 *μ*m and (bottom) 12 *μ*m. See also Supplemental Movies S5 and S6.

**Figure 5:**
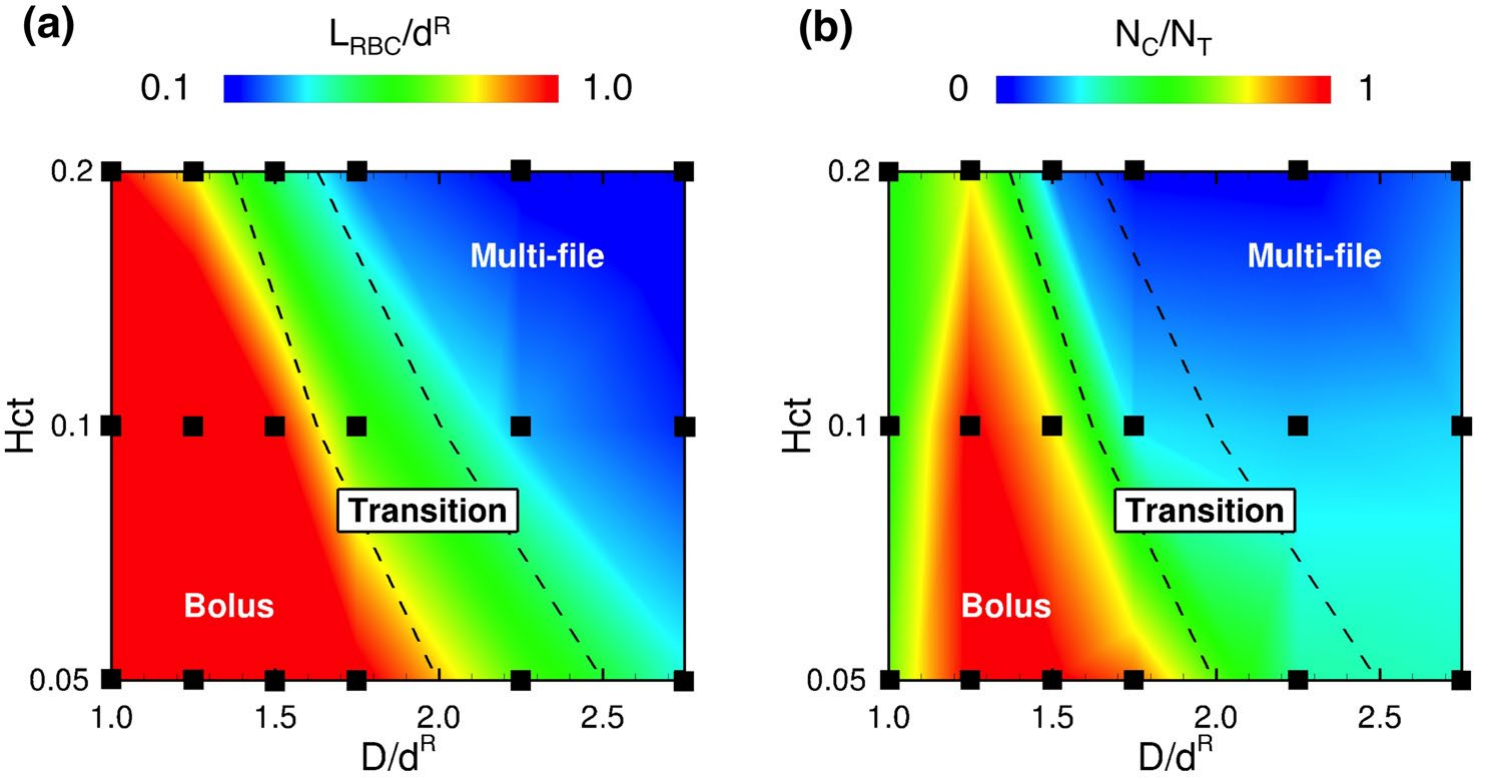
(**a**) Average distance between two RBCs normalized by the diameter of RBCs, *L*_*RBC*_/*d* ^*R*^, and (**b**) capture ratio as functions of *D*/*d* ^*R*^ and *Hct*. “Transition” refers to a state that bolus flow and multi-file flow coexists in a microvessel.

It is expected that the capture event greatly depends upon the size of plasma spaces between RBCs, and hence, the capture ratio should be affected by *Hct*. To clarify the effect of *Hct* on the capture ratio, we simulated dilute *Hct* conditions (*Hct* = 0.1 and 0.05). At *Hct* = 0.1, bolus flow occurred with enlarged plasma spaces for *D* = 10 *μ*m and 12 *μ*m, resulting in an enhancement in the capture ratio (Fig. 4a; see also Supplemental Movie S5). At *Hct* = 0.05, the size of the plasma spaces further increased, and all the MPs were stably captured (Fig. 4b; see also Supplemental Movie S6). The average distance between two RBCs (the volume centroid distance), and the capture ratio are summarized in Fig. 5a,b, respectively, as functions of *Hct* and the vessel diameter. The average distance increased when RBCs formed bolus flow, and the capture ratio was enhanced as *Hct* was decreased. Figure 5b also shows that the capture ratio peaked at *D*/*d* ^*R*^ = 1.25 (*D* = 10 *μ*m) for all values of *Hct* investigated.

### Effect of shear rate on capture ratio

**Figure 6:**
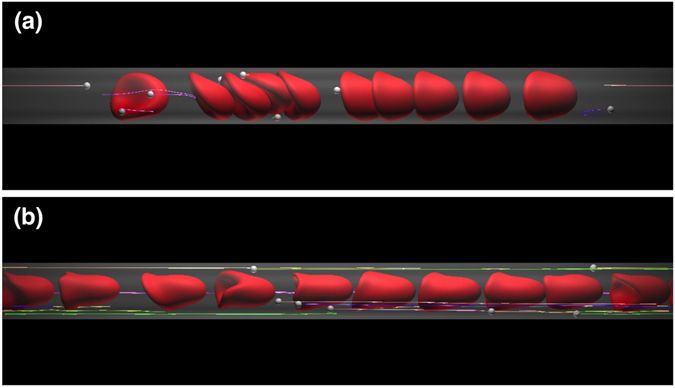
Snapshots of the flow of MPs and RBCs for (**a**) *Ca* = 0.05, and (**b**) 0.4 in capillary with a diameter of *D* = 8 *μ*m. See also Supplemental Movie S7.

**Figure 7:**
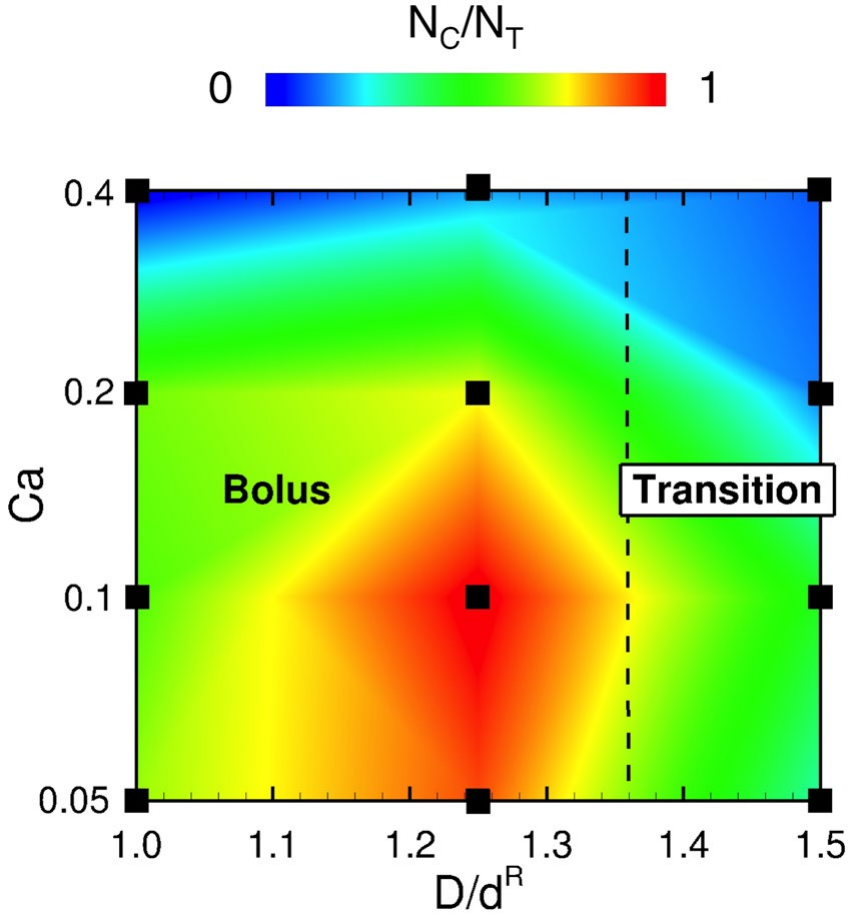
Capture ratio as functions of *D*/*d* ^*R*^ and *Ca*.

For a comprehensive understanding of capture events in bolus flow, we also investigated the effect of shear rate on the capture ratio for *Hct* = 0.2 in capillary-sized microvessels. Snapshots are shown in Fig. 6 (see also Supplemental Movie S7). For the lowest shear rate, *Ca* = 0.05, corresponding to the venule shear rate^37^, parachute-shaped RBCs underwent bolus flow at *D* = 8 *μ*m (Fig. 6a). For the highest shear rate, *Ca* = 0.4, the RBCs deformed into bullet-like shapes, similar to the RBC shape observed both in rat mesenteric microvessels^25^ and glass microcapillaries^28, 30^. Because the elongation of RBCs at higher shear rates reduces the size of plasma spaces between RBCs, MPs were marginated as shown in Fig. 6b. The effect of shear rate on the capture ratio was summarized in Fig. 7. The capture ratio was maximized at *D*/*d* ^*R*^ = 1.25 (*D* = 10 *μ*m) independent of shear rate, but was reduced when the shear rate increased.

## Discussion

The margination of MPs and platelets in blood has been widely investigated by *in vivo* ^5, 6^ and *in vitro* experiments^7, 8^, as well as numerical simulations^11–18^. These previous studies have concluded that MPs can more effectively approach the microvessel wall than nanoparticles because of margination. However, it has been unclear whether these results can be extended to capillaries, which are crucial sites for therapeutic drug transport.

We demonstrated that MPs were not marginated in the capillaries (*D*/*d* ^*R*^ ≤ 1.25), but captured in the bolus flow of RBCs. A few previous studies also examined capillary-sized microchannels. Müller *et al*.^12, 13^ simulated the behavior of MPs in a two-dimensional capillary with a width *W* = 10 *μ*m and reported that MPs were marginated in the capillary model. However, their RBC model was also small, and the capillary width relative to the RBC diameter was *W*/*d* ^*R*^ = 1.64. This width likely promoted multi-file flow rather than bolus flow (Fig. 5). Krüger^11^ also investigated the behavior of platelets in a capillary with a diameter of 10 *μ*m (*D*/*d* ^*R*^ = 1.25). The author examined a relatively higher *Hct* value (*Hct* ≈ 0.37) than physiological values in capillaries (*Hct* ≤ 0.2^37^), and found that RBCs formed multi-file flow, resulting in platelet margination at a concentrated RBC condition. Thus, the previous view that MPs efficiently approach microvessel walls^9, 12, 13, 20^ may only apply to relatively large microvessels and not to capillaries. Our results suggest that MPs could be rather inefficient drug carriers in capillaries because of capture events, and that nanoparticles, which are more randomly distributed in the capillaries, could be more effective carriers.

In this study, MPs were modeled as capsules less deformable than RBCs. Deformable particles experience lift forces perpendicular to the wall, and these lift forces may affect capture events. Although we think that capture events are mainly dominated by particle size, it is not clear whether the present results are straightforwardly applicable to rigid microparticles. Therefore, it should be noted that the present results are most applicable to cases in which the capillary number of MPs ($C{a}^{P}=\mu \dot{\gamma }{d}^{P}\mathrm{/2}{G}_{s}^{P}$) is larger than 10^−3^. MPs were also modeled as non-Brownian particles. Using the Stokes-Einstein equation, the diffusion coefficient of 1 *μ*m particles is estimated as *D*_*P*_ ≈ 3.8 × 10^−13^ m^2^/s. The Peclet number, $Pe=\dot{\gamma }{d}^{P2}\mathrm{/4}{D}_{P}$, then ranges from 28 to 220. In addition, RBC flow induces stronger dispersion than Brownian diffusion^38^. Thus, Brownian diffusion would have a small effect on this problem.

Recently, various types of microfluidic devices have been developed for human blood samples, and microparticles have been often used for micro-PIV and micro-PTV measurements of blood flow^39, 40^. Because of the relatively large sizes of microfluidic devices, this capture event has not been reported even in microfluidics studies. In the case of liquid drops, Ohmura *et al*.^41^ recently reported capture events in a microfluidic device, in which small droplets were captured between successive large droplets. We hope that this capture phenomenon can be confirmed in blood flow experiments in the near future.

In summary, MPs with a diameter of 1 *μ*m were not marginated under physiologically relevant *Hct* conditions in capillaries, but instead captured by the bolus flow of RBCs. Once the MPs were captured, they rarely escaped from the vortex-like flows between RBCs. These results suggest that microparticles are disadvantageous drug carriers for targeting capillary districts, so nanoparticles may be more effective carriers.

## Methods

### Flow and cell models

Consider a cellular flow consisting of plasma, RBCs, and MPs in microvessels ranging from 8 *μ*m to 22 *μ*m in diameter. Hereafter, the superscripts *R* and *P* represent parameters for RBCs and MPs, respectively. The microvessels were modeled as a cylindrical vessel of diameter *D*. The length of the computational domain was approximately 100 *μ*m, and periodic boundary conditions were employed in the flow direction to observe the long-term behaviors of MPs. An RBC was modeled as a biconcave capsule, or a Newtonian fluid enclosed by a thin elastic membrane, with a major diameter *d* ^*R*^ = 8 *μ*m, and maximum thickness *t* ^*R*^ = 2 *μ*m.

The membrane follows the Skalak constitutive law^42^:

1 $${w}_{s}=\frac{{G}_{s}}{4}({I}_{1}^{2}+2{I}_{1}-2{I}_{2}+C{I}_{2}^{2})$$

where *w*_*s*_ is the strain energy density function, *G*_*s*_ is the surface shear elastic modulus of the cell membrane, and *C* is a coefficient representing the area incompressibility. The surface shear elastic modulus and area incompressibility coefficient of RBCs were determined to be ${G}_{s}^{R}=4.0$ *μ*N/m and *C* ^*R*^ = 10^2^, respectively^43^. Bending resistance was also considered^44^, with a bending modulus ${k}_{b}^{R}=5.8\times {10}^{-19}$ N · m^45^. The viscosity of cytoplasm was taken to be *μ* ^*R*^ = 6.0 × 10^−3^ Pa · s, which is five times higher than the viscosity of plasma (*μ* = 1.2 × 10^−3^ Pa · s). These values successfully reproduced various behaviors of RBCs, including the deformation of RBCs in shear flow and the thickness of cell-depleted peripheral layer (CDPL)^43^.

An MP was modeled as a small spherical capsule with a diameter *d* ^*P*^ = 1 *μ*m. An experiment using atomic force microscopy showed that the deformability of human platelets is generally lower than RBCs^46^. Here, MPs were modeled as less deformable capsules than RBCs; *i.e*., the membrane shear elasticity was 10 times larger than RBCs ($R{G}_{s}={G}_{s}^{P}/{G}_{s}^{R}=10$), while the other parameters remained the same (*i.e*., *C* ^*P*^ = *C* ^*R*^, *μ* ^*P*^ = *μ* ^*R*^, and ${k}_{b}^{P}={k}_{b}^{R}$).

### Numerical simulation

Various types of numerical methods have been developed for simulating cellular flow^36, 47^. In this study, we used the Lattice-Boltzmann method (LBM)^48, 49^ coupling with the finite element method (FEM)^50^. The membrane mechanics was solved by FEM^50^, given by

2 $${\int }_{S}\hat{{\boldsymbol{u}}}\cdot {\boldsymbol{q}}dS={\int }_{S}\hat{{\boldsymbol{\varepsilon }}}:{\boldsymbol{T}}dS,$$

where ***T*** is the Cauchy stress tensor, ***q*** is the load on the membrane, $\hat{{\boldsymbol{u}}}$ is the virtual displacement, and $\hat{{\boldsymbol{\varepsilon }}}$ is the virtual strain. The fluid mechanics was solved by LBM^48^, *i.e*.,

3 $${f}_{i}({\boldsymbol{x}}+{{\boldsymbol{c}}}_{i}{\rm{\Delta }}t,t+{\rm{\Delta }}t)-{f}_{i}({\boldsymbol{x}},t)=-\frac{1}{\tau }[{f}_{i}({\boldsymbol{x}},t)-{f}_{i}^{eq}({\boldsymbol{x}},t)]+{F}_{i}{\rm{\Delta }}t,$$

where *f*_*i*_ is the particle distribution function for particles with velocity ***c***_*i*_ at a position ***x***, Δ*t* is the time step size, ${f}_{i}^{eq}$ is the equilibrium distribution, *τ* is the nondimensional relaxation time, and *F*_*i*_ is the external force term. The LBM D3Q19 lattice model was used. FEM and LBM were coupled by the immersed boundary method^51^. The volume-of-fluid (VOF) method^52^ and front-tracking method^53^ are also employed to update the viscosity in the fluid mesh. All procedures were fully implemented on graphics processing unit (GPU) to accelerate the numerical simulation^54^. Our coupling method has been successfully applied to numerical analyses of leukocytes^43^, circulating tumor cells^55^ and cell adhesion^56^. The solid and fluid mesh sizes were set to be 250 nm (an unstructured mesh with 5,120 elements was used for the RBC membrane and 1,280 elements for the MP membrane). In the case of the smallest capillary (*D* = 8 *μ*m), mesh sizes were set to be 125 nm for the LBM and FEM meshes (20,480 elements for RBCs and 5,120 elements for MPs).

The shear rate was defined as $\dot{\gamma }={U}_{m}/D$, where *U*_*m*_ is the mean fluid velocity in the absence of cells under the same pressure gradient. We focused on a shear rate of $\dot{\gamma }=167$ s^−1^ (wall shear rate ${\dot{\gamma }}_{w}\mathrm{=1},336$ s^−1^) and an RBC volume fraction of *Hct* = 0.2, to simulate a physiologically relevant *Hct* in capillaries^37^. The shear rate can be normalized to form the capillary number $Ca=\mu \dot{\gamma }{d}^{R}\mathrm{/2}{G}_{s}^{R}$, such that $\dot{\gamma }=167$ s^−1^ corresponds to *Ca* = 0.2. The volume fraction of MPs was set to 0.001, corresponding to the volume fraction of platelets in human blood^57, 58^. We only used data after the thickness of CDPL reached a plateau (this time is referred to as *t* = 0, and defined as quasi-steady state) to reduce the influence of the initial conditions. Time-averaging was performed for a time period of 1.0 s to 1.5 s. Total number of MPs and RBCs in our simulations are listed in Table 1.

**Table 1:** Total number of MPs and RBCs.

*D* [*μ*m]	Total number of MPs (*N*_*T*_)	Total number of RBCs		
		*Hct* = 0.2	*Hct* = 0.1	*Hct* = 0.05
8	10	10	5	3
10	18	16	8	4
12	24	22	11	5
14	32	32	16	8
18	64	52	26	14
22	94	78	40	20

## Electronic supplementary material

Supplemental Movies

Movie S1

Movie S2

Movie S3

Movie S4

Movie S5

Movie S6

Movie S7
