# Supplementary material for: Capture of microparticles by bolus flow of red blood cells in capillaries
Source: Sci Rep. 2017 Jul 14;7:5381. doi: 10.1038/s41598-017-05924-7 (PMC5511268; doi:10.1038/s41598-017-05924-7)
Supplement: Supplementary file 1 — Supplemental Movies [file 41598_2017_5924_MOESM1_ESM.pdf]

Capture of microparticles by bolus flow of red blood cells in capillaries

Naoki Takeishi and Yohsuke Imai

Supplemental Movies

Supplemental Movie S1: The flow of MPs and RBCs for *Hct* = 0.2 and *Ca* = 0.2 in microvessels with a diameter *D* = 8 µm.

Supplemental Movie S2: The flow of MPs and RBCs for *Hct* = 0.2 and *Ca* = 0.2 in microvessels with a diameter *D* = 10 µm.

Supplemental Movie S3: The flow of MPs and RBCs for *Hct* = 0.2 and *Ca* = 0.2 in microvessels with a diameter *D* = 12 µm.

Supplemental Movie S4: The flow of MPs and RBCs for *Hct* = 0.2 and *Ca* = 0.2 in microvessels with a diameter *D* = 22 µm.

Supplemental Movie S5: The flow of MPs and RBCs for *Hct* = 0.1 and *Ca* = 0.2 in microvessels with diameters *D* = 10 µm (top) and *D* = 12 µm (bottom).

Supplemental Movie S6: The flow of MPs and RBCs for *Hct* = 0.05 and *Ca* = 0.2 in microvessels with diameters *D* = 10 µm (top) and *D* = 12 µm (bottom).

Supplemental Movie S7: The flow of MPs and RBCs at *Hct* = 0.2 in a microvessel

with a diameter D = 8 μm for Ca = 0.05 (top) and Ca = 0.4 (bottom).
